# Section on Medical Jurisprudence, Physiology, and Hygiene

**Published:** 1866-07

**Authors:** 


SECTION ON MEDICAL JURISPRUDENCE, PHYSIOLOGY, AND HYGIENE.

*First Session, May 1, 1866.*

The session was organized at Concordia Hall, 3 P.M., May 1, by electing Dr. Wilson Jewell, of Pennyslvania, Chairman; and Dr. A. N. Bell, of New York, Secretary.

